# Validity of a quantitative tool of trunk asymmetry based on digital photographs in patients with idiopathic scoliosis

**DOI:** 10.1186/1748-7161-9-S1-O6

**Published:** 2014-12-04

**Authors:** Antonia Matamalas, Joan Bago, Elisabetta DAgata, Ferran Pellise

**Affiliations:** 1 Hospital Vall Hebron, Barcelona, Spain

## Background

Some measures of trunk deformity obtained in digital photography may be useful in the assessment of trunk deformity. The relationships between these measures and radiographic indexes have not been fully analyzed.

## Aim

To assess the reliability and validity of a clinical assessment tool of the trunk deformity based on photographs as compared to radiological measurements.

## Study design

Cross-sectional study. Concurrent validity between postural indexes obtained from digital photographs and radiographs.

## Methods

Front and back digital photographs of patients with idiopathic scoliosis (Cobb>25º) were obtained. Shoulder, armpit and waist angles, in addition to trunk asymmetry indexes, were calculated on front and back photographs with Surgimap software. On AP radiographs Coronal Cobb angles and radiological shoulder imbalance using CRIA angle (Angle between horizontal line and a line drawn between, the right and left intersection points of clavicle and rib cage) were calculated. Intra-class correlation coefficient was used to assess intra and inter-rater reliability. The Pearson correlation coefficients (r) were used to estimate concurrent validity between both methods.

## Results

80 patients (68 females) mean age 20.3 years (12-40 years) were included. Mean Cobb Maximum (CobbMax) was 45.9º (25.1º-77.2º).All measures had a good to excellent intra and inter-rater reliability both in front and back photographs. Waist height angle and CobbMax angle, both in front and back photographs, were significantly correlated (r=0.42 back/r=0.29 front view). There was no significant correlation between proximal thoracic curve magnitude and any of the shoulder measures. The correlation between shoulder and armpit height angles and radiographic clavicle tilt were -0.44 and -0.41 respectively on frontal view. There was a correlation between trapezium angle ratio and clavicle tilt in both views (r=0.43 back/r=0.32 front view). No other statistically significant correlations between both methods were found.

## Conclusions

Digital photography measurement of waist height is useful to assess trunk deformity in idiopathic scoliosis due to a good correlation with Cobb. Shoulder asymmetry indexes like shoulder height angle can be clinical measures of shoulder imbalance. Trunk asymmetry indexes are not correlated with radiological measures.

